# Safety, Efficacy and Attributes of 2.5% Selenium Sulfide Shampoo in the Treatment of Dandruff: A Single-Center Study

**DOI:** 10.7759/cureus.57148

**Published:** 2024-03-28

**Authors:** Gauri Godse, Kiran Godse

**Affiliations:** 1 Dermatology, Shree Skin Centre, Navi Mumbai, IND; 2 Dermatalogy, Dr. D. Y. Patil Medical College and Hospital, Navi Mumbai, IND

**Keywords:** product attributes, scalp itch, sebum, indian participants, pityriasis capitis, selenium sulfide

## Abstract

Background: Selenium sulfide, available as a shampoo or topical lotion at 1%, or 2.5% concentrations in India, is used as a topical antiseborrheic and antifungal for the treatment of dandruff, seborrheic dermatitis, psoriasis, and tinea versicolor. In the present study, the safety, efficacy, and attributes of 2.5% selenium sulfide shampoo were evaluated in Indian participants with dandruff.

Methods: A single-center, single-arm, prospective, investigator-initiated, open-label, post-marketing interventional study was conducted on Indian subjects aged 18-70 years diagnosed with moderate dandruff who were prescribed 2.5% selenium sulfide shampoo every three days for four weeks. The primary endpoints were 1) reduction in total dandruff score assessed using a clinical grading scale for adherent and loose dandruff from baseline to weeks 1, 2, and 4, and 2) incidence of adverse events up to the end of the study. The key secondary endpoints were 1) participants’ perception of shampoo attributes (dandruff reduction, scalp itch, scalp oiliness/greasiness, or fragrance) as assessed by a subjective self-assessment questionnaire post-first wash and at weeks 1, 2, and/or 4; 2) satisfaction with treatment as assessed by investigators and participants using a subjective self-assessment questionnaire at week 4; and 3) reduction in scalp sebum as assessed with a meibometer at weeks 2 and 4. Statistical analysis was performed using the Wilcoxon signed-rank test for continuous variables and the Chi-square test for categorical variables. A p-value of 0.05 was considered to be statistically significant.

Results: Of 34 enrolled subjects, 30 completed the four-week study. The mean (standard deviation, SD) age of the study participants was 29.8 (7.87) years, with the majority being females (n=18; 60.0%). Mean (SD) total dandruff score significantly (p=0.001) reduced from a baseline score of 11.5 (2.15) to 7.17 (2.12) at week 1, 4.93 (1.72) at week 2, and 2.5 (1.17) at week 4. All the participants reported dandruff reduction and acceptable fragrance of the shampoo at four weeks. Absence of itching and reduction in oiliness was reported by 73.3% (n=22) of participants at week 4 and by 50.0% (n=15) of participants at week 2, respectively. All participants reported good, very good, or excellent satisfaction with the test shampoo at week 4, whereas the investigators rated the shampoo as very good or excellent in managing dandruff in all participants. At week 4, erythema was reported to be absent in all participants. No adverse events were reported during the study.

Conclusions: The 2.5% selenium sulfide shampoo was found to be effective in the management of dandruff and related symptoms like itching, oiliness, and greasiness and had a good safety profile in Indian participants with dandruff.

## Introduction

Pityriasis capitis, commonly known as dandruff, manifests as a scaly scalp condition [1]. Dandruff is considered a mild degree of seborrheic dermatitis [2]. The individual may experience a sense of self-consciousness and embarrassment. The presence of dandruff can lead to embarrassing itching as well. The impact of dandruff on individuals goes beyond medical concerns and leads to numerous social and psychological challenges [1]. Various studies have shown that dandruff affects about 50% of the global population, with men being more prone to develop dandruff than women [1].

Skin biocenosis, in particular, is caused by Malassezia spp. flora plays a key etiologic role in the onset and development of dandruff [1]. Other etiologic factors are sebum secretions and personal susceptibility. Malassezia spp. lack fatty acid synthase genes; hence, they satisfy their obligate need for fatty acids by secreting multiple lipases that metabolize triglycerides in sebaceous lipids on the scalp surface to release free fatty acids [3,4]. These free fatty acids also act as skin irritants and induce inflammatory responses that are typical of dandruff [5].

Dandruff and its symptoms are usually treated with topical antifungal formulations to eradicate Malassezia spp. from the scalp [6]. Some widely used and effective antifungal agents include selenium sulfide, salicylic acid, and imidazole antifungals like ketoconazole, zinc pyrithione, and ciclopirox, as wash-off formulations [7].

Selenium sulfide is used to treat dandruff, seborrheic dermatitis of the scalp, and tinea capitis [6]. It appears to have a cytostatic activity on the epidermis and follicular epithelium that reduces corneocyte production and subsequent flaking. The antimitotic action of topical selenium sulfide leads to a decrease in the turnover of epidermal cells [6]. Furthermore, it possesses antibacterial, antiseborrheic, and mild antifungal properties that potentially augment its efficacy [7]. A previous study by Wu et al. suggested that the antifungal action of selenium sulfide is attributed to the cellular oxygen-eliminating system of the fungi, which leads to an increased production of intracellular reactive oxygen species (ROS) [7,8]. In a recent study, selenium sulfide-containing shampoo was found to possess the highest cytostatic and keratolytic activities among four types of shampoos used in the management of dandruff [9]. Shampoos, being the most frequently prescribed treatment for the hair and scalp, offer increased efficacy of anti-dandruff agents, allowing shorter contact time, and reducing irritation [10].

The objectives of this study were to evaluate the safety and efficacy of 2.5% selenium sulfide shampoo, along with investigators’ and participants’ satisfaction with dandruff clearance, and participants’ perception of the shampoo attributes in Indian subjects with moderate dandruff.

## Materials and methods

Study design

A single-center, single-arm, prospective, investigator-initiated, open-label, postmarketing, interventional study was conducted at Shree Skin Centre, Navi Mumbai, India, from December 2022 to April 2023. The study protocol was reviewed and approved by the Royal Pune Independent Ethics Committee (ECR/45/Indt/MH/2013/RR-19). The study was registered with the Clinical Trials Registry of India on 17 October 2022 (CTRI/2022/10/046553) and conducted in compliance with the protocol, Declaration of Helsinki, Good Clinical Practice Guidelines, and New Drugs and Clinical Trials Rules, 2019. Written informed consent was obtained from all participants before enrollment.

Eligibility criteria

Adult men or women with moderate dandruff (score> 10) as assessed using a clinical grading scale, aged 18-70 years, who were willing to comply with the study specifications and refrain from the use of any other topical medications that would affect the results of the trial, including medicated shampoos/oils, or antibiotics, during the treatment and relapse periods, were included in the study [11,12]. Exclusion criteria were pregnant or lactating women; subjects with a history or presence of compromising dermatosis elsewhere on the skin, Parkinson's disease, human immunodeficiency virus infection, or infections or disorders of the central nervous system; subjects with actinically damaged skin; subjects who had used an antidandruff agent in the 14 days before enrolment or those with any skin condition that would interfere with the diagnosis or assessment of dandruff (e.g., psoriasis, acne, or atopic dermatitis); subjects with clinically significant systemic disease (e.g., immunological deficiencies, acquired immunodeficiency syndrome, current malignancies, or uncontrolled diabetes); subjects who had used systemic antifungals, steroids, antibiotics, anti-inflammatory agents, retinoids or cytostatic or immunomodulating drugs (e.g., cyclosporine, tacrolimus, or pimecrolimus) within one month before enrollment; subjects who had used antihistamines or topical steroids, retinoids, anti-inflammatory agents, antibiotics, or treatment for adherent dandruff (e.g., coal tar preparation, antidandruff shampoo/oils/gels/creams/conditioners) within two weeks before enrollment; and subjects participating in or who were in the exclusion period of a similar cosmetic or therapeutic trial.

Study interventions

The patients were asked to apply 5 to 10 mL of the 2.5% selenium sulfide shampoo to wet the scalp and massage it. After Leaving it on for two to three minutes, they were asked to rinse thoroughly and repeat. The patients were prescribed 2.5% selenium sulfide shampoo twice weekly for four weeks.

Study endpoints

The primary endpoints were 1) dandruff reduction assessed by adherent and loose dandruff scores based on a clinical grading scale at baseline, and weeks 1, 2, and 4; and 2) incidence of adverse events reported anytime during the study. The secondary endpoints were 1) participant-reported shampoo attributes (fragrance and dandruff reduction, scalp itchiness, and scalp oiliness/greasiness) assessed using a subjective self-assessment questionnaire after the first wash and at weeks 1, 2, and/or 4; 2) investigator-reported satisfaction with treatment assessed using a subjective self-assessment questionnaire at weeks 1, 2, and/or 4; 3) reduction in scalp sebum as assessed with a meibometer, from baseline to weeks 2 and 4; and 4) participant-reported satisfaction with treatment assessed using a subjective self-assessment questionnaire at week 4 [13].

Study assessments

Clinical Grading of Dandruff Reduction

The scalp was marked into six zones as follows: frontal right and left, temporal right and left, and occipital right and left. The investigators graded dandruff in all zones at baseline and weeks 1, 2 and 4 using the clinical grading scale by Danby et al. as follows: 0 = no dandruff, 1-2 = almost no/slight dandruff, 3-4 = mild dandruff, 5-6 = moderate dandruff, 7-8 = marked dandruff, and 9-10 = severe/heavy dandruff [12]. The total score was calculated as the sum of the scores of all six zones.

Reduction in Sebum

Meibometer MB 560 (Courage + Khazaka electronic GmbH S/N: 21345997; software details: MPA CTplus-1.1.4.6 Databse-1.1.4) was used to determine sebum concentration at baseline and weeks 2 and 4 [13]. Meibometer has a photometric measuring unit consisting of a light emitter and receiver, which are situated opposite to each other inside the device. A matt synthetic foil with a rough surface becomes transparent when in contact with lipids. The higher the lipid content, the higher the transparency of the foil. The device measures the transparency of the foil after contact with the lipid contacting fluid. A test area on the scalp was identified by the investigators and used for the evaluation of sebum at all study visits. A Meibo strip was fixed onto a pen-like holder, placed onto the specific scalp area for 30 seconds, and loaded into the Meibometer for measurement of sebum arbitrary units. One measurement per participant at each visit.

Assessment of Investigator-Reported Satisfaction

The investigators rated erythema on each patient’s scalp as absent, mild, moderate or severe at baseline and weeks 1, 2, and 4. In addition, the investigators also rated the overall effect of the shampoo in managing dandruff as excellent, very good, good, or poor at week 4.

Assessment of Participant-Reported Satisfaction and Perception

Each participant was asked to fill out a subjective questionnaire (Appendices, Table 5) to assess their satisfaction with the shampoo and their perception of its attributes, such as fragrance and the ability to reduce dandruff, scalp itchiness, and scalp oiliness.

Statistical analyses

No formal sample size calculation was performed as this was a pilot, exploratory study; however, a sample size of 34 subjects was thought to be appropriate for evaluating the study objectives. Continuous variables were summarized as n, mean, and standard deviation (SD). Categorical variables were summarized as n and percentages. Wilcoxon signed rank test and Chi-square test were used to determine statistical significance between baseline and post-baseline visits for continuous and categorical variables, respectively. A p-value of <0.05 was considered to be statistically significant. Statistical analyses were done using SPSS for Windows, Version 10.0. (SPSS Inc., Chicago).

## Results

Subject disposition and baseline characteristics

**Figure 1:**
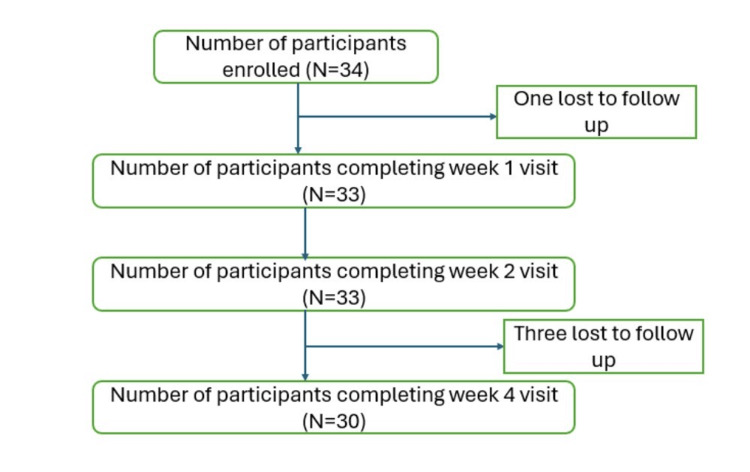
Subject disposition

Out of 34 enrolled subjects, 30 completed the study in four weeks (Figure 1). Twelve (40.0%) of the participants were men, and 18 (60.0%) were women. Mean (SD) age of the study participants was 29.8 (7.87) years.

In all, 18 protocol deviations were reported by 17 participants, all of which were related to delays in study visits ranging from three to 14 days. None of these deviations were deemed by the investigators to affect any of the study outcomes. One participant was prescribed ivermectin 12 mg and albendazole 400 mg concomitant treatment once in seven days for one month for the treatment of pediculosis, which was not deemed by the investigators to have any effect on the study.

Reduction in dandruff scores

**Figure 2:**
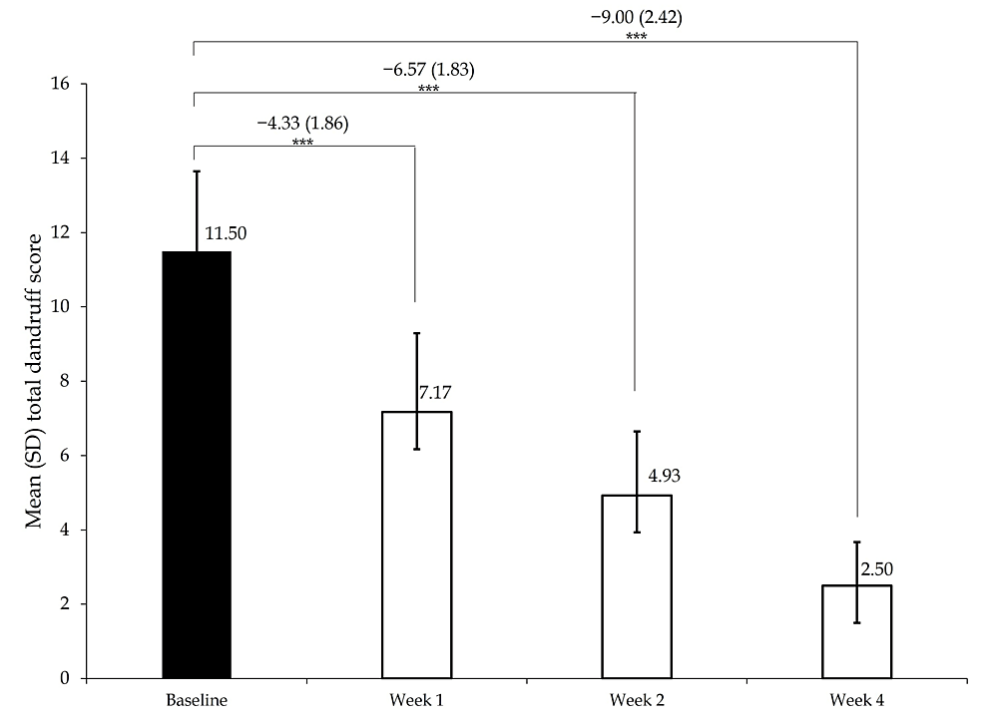
Change in dandruff score SD, standard deviation. P value by Wilcoxon signed-rank test.

Figure 2 shows the mean (SD) total dandruff score at baseline and follow-up visits at weeks 1, 2, and 4. The mean (SD) score decreased by 4.33 (1.86), 6.57 (1.83), and 9.0 (2.42) from baseline to weeks 1, 2, and 4, respectively (p=0.001). Thus, there was a decrease of 37.7%, 57.1%, and 78.3% in dandruff scores at weeks 1, 2, and 4, respectively, indicating a significant reduction in dandruff with regular use of the 2.5% selenium sulfide shampoo.

Participants’ perception about shampoo attributes

Ability to Reduce Scalp Itching

After 1, 2 and 4 weeks of treatment, 10.0% (n=3), 43.3% (n=13), and 73.3% (n=22) of participants reported absence of scalp itching, respectively, compared with 6.7% (n=2) of participants at baseline (Table 1). The differences in proportions were significant at weeks 2 and 4 (p=0.001). None of the participants reported moderate or severe itching at any of the post-baseline visits.

**Table 1:** Participant-reported severity of scalp itching P value by Chi-square test.

Proportion of participants, n (%)	Baseline (N = 30)	Week 1 (N = 30)	Week 2 N = 30)	Week 4 (N = 30)
Absent	2 (6.7)	3 (10.0)	13 (43.3)	22 (73.3)
Mild	19 (63.3)	27 (90.0)	17 (56.7)	8 (26.7)
Moderate	9 (30.0)	-	-	-
Severe	-	-	-	-
P value	-	0.640	0.001	0.001

Post-first Wash Experience

After the first wash with shampoo, 90.0% (n=27) of participants reported reduction in itching of scalp, 63.3% (n=19) reported reduction in flaking, and only 6.7% (n=2) reported no change in either itching or flaking (Table 2). The mean (SD) score for feel of hair after first wash was 2.67 (0.48), indicating (good to very good feel. The mean (SD) score for rinsibility was 2.97 (0.41), indicating nearly easy rinsibility.

**Table 2:** Participants’ post-first wash experience SD, standard deviation.

Parameter, n (%)	Overall (N = 30)
Reduction in itching	27 (90.0)
Reduction in flaking	19 (63.3)
No change in itching or flaking	2 (6.7)
Hair feel score, mean (SD)	2.67 (0.48)
Shampoo rinsibility score, mean (SD)	2.97 (0.41)

Participants’ Perception of Other Shampoo Attributes

In all, 90.0% (n=27) and 96.7% (n=29) of participants agreed that there was a reduction in dandruff, at weeks 1 (within two washes) and 2 (within four washes), respectively. At week 4, all participants agreed or strongly agreed that there was a reduction in dandruff (Table 3).

**Table 3:** Participants’ perception of the reduction in dandruff and oiliness or greasiness of the scalp NA, not assessed.

Proportion of participants, n (%)	Baseline (N = 30)	Week 1 (N = 30)	Week 2 (N = 30)	Week 4 (N = 30)
Reduction in dandruff				
Strongly agree	-	-	-	2 (6.7)
Agree		27 (90.0)	29 (96.7)	28 (93.3)
Neither agree nor disagree	-	3 (10.0)	1 (3.3)	
Disagree	-	0 (0.0)	0 (0.0)	0 (0.0)
Strongly disagree	-	0 (0.0)	0 (0.0)	0 (0.0)
Reduction in oiliness and greasiness				
Strongly agree	-	NA	0 (0.0)	NA
Agree	-	NA	15 (50.0)	NA
Neither agree nor disagree	-	NA	13 (43.3)	NA
Disagree	-	NA	2 (6.7)	NA
Strongly disagree	-	NA	0 (0.0)	NA

After two weeks of wash, 50.0% (n=15) of participants agreed that there was a reduction in oiliness and greasiness of the scalp (Table 3). At week 4, 96.7% (n=29) of participants reported that the shampoo was very good or excellent in managing dandruff, while 3.3% (n=1) reported that it was good. Moreover, at week 4, all participants reported that the shampoo had an acceptable fragrance, that they would prefer to use this shampoo again in the future for managing dandruff, and that they would recommend it to others for managing dandruff.

Investigator satisfaction with treatment

According to the investigator's assessment, 86.7% (n=26) had mild erythema at baseline. This proportion significantly decreased to 33.3% (n=10), 3.3% (n=1), and 0.0% at weeks 1, 2, and 4, respectively (p=0.001; Table 4). Thus, none of the participants had erythema by the end of week 4.

**Table 4:** Investigator-reported change in erythema P value by Chi-square test.

Proportion of participants, n (%)	Baseline (N = 30)	Week 1 (N = 30)	Week 2 (N = 30)	Week 4 (N = 30)
Absent	-	20 (66.7)	29 (96.7)	30 (100)
Mild	26 (86.7)	10 (33.3)	1 (3.3)	-
Moderate	4 (13.3)	-	-	-
Severe	-	-	-	-
P value	-	0.001	0.001	0.001

At week 4, the investigators also reported that the shampoo was excellent in managing dandruff in 93.3% (n=28) of participants and very good in managing dandruff in the remaining 6.7% (n=2) of participants.

Reduction in sebum

The mean (SD) sebum score was 279.42 (144.28) at baseline, which changed to 249.52 (130.99) at week 2, and 303.50 (149.92) at week 4. The differences from baseline were not statistically significant (p>0.05), indicating that selenium sulfide may not have an effect on sebum secretion.

## Discussion

Selenium sulfide was first approved for medical use in 1951 [14]. Since then, it has been widely used for the treatment of seborrheic dermatitis [15]. As reported by Clark et al., selenium sulfide can control the symptoms associated with dandruff and seborrheic dermatitis at a fraction of the cost of other treatments [16]. The present study was conducted to evaluate the safety and efficacy of a shampoo containing 2.5% (USP) selenium sulfide as a topical suspension in Indian patients with dandruff. 

The primary endpoint of reduction in total dandruff scores assessed using a clinical grading scale was met, as was evident from the reduction in total scores from baseline to weeks 1, 2, and 4. This efficacy of selenium sulfide (2.5%) could be attributed to its antifungal, cytostatic, and keratolytic effects [6]. In a study by Davies et al., the reduction in total dandruff severity scores was 57.14% (2.1 to 0.9) across 29 days, which was lower than the current study where selenium sulfide 2.5% showed a reduction of 78.3% across four weeks [17]. In a recent study, selenium sulfide 2.5% shampoo was found to have higher cytostatic and keratolytic activities and lower cytotoxic activity than shampoos containing 2% ketoconazole, 1% zinc pyrithione, or 2% ketoconazole + 1% zinc pyrithione [9]. The efficacy of 2.5% selenium sulfide was also confirmed in a 3-arm, randomized controlled trial involving 246 patients with moderate to severe dandruff who received shampoos containing 2.5% selenium sulfide, 2% ketoconazole, or placebo [12]. The results of the present study and previous reports indicate that 2.5% selenium sulfide could be an effective standalone treatment for moderate to severe dandruff.

Antifungals such as azoles are commonly used in the treatment of moderate to severe dandruff. However, a few recent studies have identified Malassezia species with resistance to azole antifungals. The resistance could be attributed to increased prophylactic use of azoles, prolonged treatment regimens, or long-term use of low-dose azoles [18]. Another study reported found increased number of ketoconazole-resistant M. restricta strains in patients with dandruff [19]. The above could therefore provide some clinical challenges in more severe fungal infections due to the presence of anti-fungal resistance [20]. The findings from the above study indicate can selenium sulfide 2.5% could be used as an efficacious non-anti-fungal treatment option in moderate to severe dandruff.

No adverse events were reported by any participant throughout the study, indicating that the 2.5% selenium sulfide-containing shampoo has a good safety profile. In the study by Danby et al., pruritus and irritation of the scalp were the two most commonly observed adverse events [12]. Other commonly used treatment options, such as ketoconazole, have been reported to cause adverse effects such as itching, burning, and contact dermatitis [21].

Patient perception is an important measure of the quality of healthcare [22,23]. After the first wash with 2.5% selenium sulfide shampoo, the majority of the participants reported a reduction in scalp itching (90%) and flaking (63.3), indicating that selenium sulfide could have a rapid onset of action and provide immediate relief in individuals suffering from dandruff and associated discomfort. Other favorable, post-first wash, participant-reported outcomes, such as improvement in hair feel and ease of rinsibility of the shampoo could further enhance individual experience and increase its acceptability among subjects with dandruff [24]. In all, 90.0% of participants agreed that there was a reduction in dandruff within two washes, and 96.7% of participants agreed that there was a reduction in dandruff within four washes. Another similar study evaluating the efficacy of tea tree oil shampoo reported a modest improvement of 23% in symptoms of itching [25].

An increasing proportion of participants reported a reduction in itching and dandruff over the four-week study duration. Moreover, half the participants agreed that there was a reduction in oiliness and greasiness of the scalp at the end of two weeks. The previous study evaluating tea tree oil shampoo reported an improvement of 25.9% in the greasiness score, which was lower than the present study [25]. While the extent of oiliness and greasiness could be associated with the amount of sebum present on the scalp, no significant reduction in sebum concentration was observed with Meibometer analysis at two or four weeks after treatment. Previous studies in ketoconazole have also reported no reduction in sebum levels. Dobrev et al. reported that ketoconazole does not reduce sebum levels but improves its delivery to the skin surface. Non-reduction of sebum due to selenium sulfide could be attributed to a similar reason. A more efficient flow of sebum thereby contributes to the reduction of yeast and bacteria colonizing the pilosebaceous duct [26].

All participants reported that the shampoo has an acceptable fragrance, that they would prefer using the shampoo in managing dandruff again, and that they would also recommend it to others. Chen et al previously reported that the odor associated with selenium sulfide might be a barrier to shampoo prescription [27]. However, the results of this study indicate that the formulation of selenium sulfide used in this study has an acceptable odor. The overall favorable feedback holds significant implications for user satisfaction with selenium sulfide that could influence its usage. Previous studies have reported that good fragrances elicit positive emotions and cause memory enhancement [28].

Patients and physicians may have different perceptions regarding therapies. Therefore, assessing physician satisfaction is as important as patient perception. The investigators reported that the shampoo was excellent in managing dandruff in the majority of the participants (93.3%) at the end of four weeks. This indicates that investigators’ and participants’ opinions were largely consistent with respect to the overall quality of the shampoo.

Dandruff is commonly associated with erythema of the scalp [29]. According to the investigators in this study, while the majority of the participants had mild erythema at baseline, the proportion of participants without erythema significantly increased at weeks 1 and 2 and by the end of the study at four weeks, none of the participants had scalp erythema.

The study had some limitations, such as the absence of a control arm, a small sample size limiting the generalizability of the study results, the possibility of recall bias, and the subjective nature of some assessments. However, this was a pilot study, and subsequent studies must include a more detailed comparative study. Nevertheless, previous pilot studies for preliminary efficacy and safety in dandruff and seborrheic dermatitis have had similar sample sizes [30,31].

## Conclusions

Results from this single-arm, single-center, prospective, investigator-initiated, post-marketing study conducted in Indian subjects with moderate dandruff indicate that the test shampoo containing 2.5% selenium sulfide as a topical suspension caused significant reduction in total dandruff scores over four weeks of treatment, had a good safety profile throughout the study, and yielded favorable investigator- and participant-reported outcomes with respect to shampoo attributes after the first wash and over four weeks of treatment. Nevertheless, further large-scale, comparative studies are warranted to corroborate the findings of this pilot study.

## Appendices

**Table 5:** Participant self-reported questionnaire for assessment of shampoo attributes and satisfaction with treatment

Baseline										
Q1	How would you grade the itching of your scalp?									
	Absent		Mild			Moderate			Severe	
Post-first wash										
Q1	Did you notice									
	Reduction in itching			Reduction in flaking				No change		
Q2	Describe the feel of your hair									
	Not good	Average			Good		Very good			Excellent
Q3	How would you grade rinsibility of the shampoo?									
	0 = Difficult to rinse	Not so easy to rinse			Fairly easy to rinse		Easy to rinse			Very easy to rinse
Week 1										
Q1	Do you believe that there was reduction in your dandruff?									
	1 = Strongly disagree	2 = Disagree			3 = Neither agree nor disagree		Agree			Strongly agree
Q2	How would you grade the itching of your scalp?									
	Absent		Mild			Moderate			Severe	
Week 2										
Q1	Do you believe that there was reduction in your dandruff?									
	1 = Strongly disagree	2 = Disagree			3 = Neither agree nor disagree		Agree			Strongly agree
Q2	Do you feel that there is reduction in oiliness and greasiness of scalp?									
	1 = Strongly disagree	2 = Disagree			3 = Neither agree nor disagree		Agree			Strongly agree
Q3	How would you grade the itching of your scalp?									
	Absent		Mild			Moderate			Severe	
Week 4										
Q1	Do you believe that there was reduction in your dandruff?									
	1 = Strongly disagree	2 = Disagree			3 = Neither agree nor disagree		Agree			Strongly agree
Q2	How would you grade the itching of your scalp?									
	Absent		Mild			Moderate			Severe	
Q3	How would you rate the ability of the shampoo to manage dandruff?									
	Excellent		Very good			Good			Poor	
Q4	Do you feel the fragrance of the medicated antidandruff shampoo was									
	Acceptable					Not acceptable				
Q5	Would you prefer using this shampoo again for managing dandruff									
	Yes					No				
Q6	Would you recommend this shampoo to others in the future ?									
	Yes					No				
